# Chrysin promotes oral ulcer healing via modulating matrix metalloproteinases and vascular endothelial growth factor in rats

**DOI:** 10.22038/ijbms.2025.86895.18772

**Published:** 2025

**Authors:** Abeer Salama, Rania Elgohary

**Affiliations:** 1 Pharmacology Department, Medical Research and Clinical Studies Institute, National Research Centre (NRC), 33 El Buhouth St., Dokki, Cairo 12622, Egypt; 2 Narcotics, Ergogenics and Poisons Department, Medical Research and Clinical Studies Institute, National Research Centre (NRC), 33 El Buhouth St., Dokki, Cairo 12622, Egypt

**Keywords:** Acetic acid, Chrysin, MMP9, Oral ulcer, Rats, VEGF

## Abstract

**Objective(s):**

Oral ulcers are a common inflammatory condition affecting the mucosal lining, often causing pain and discomfort. Chrysin is a natural flavonoid with well-documented anti-oxidant and anti-inflammatory properties. This study investigates the therapeutic effect of chrysin in an experimental model of acetic acid-induced oral ulcers in rats.

**Materials and Methods:**

To establish an oral ulcerative mucositis model, 50% acetic acid was administered to the labial fornix of the inferior incisors. Chrysin gel (1% and 2%) was applied to the oral mucosa of rats with aggravated oral ulcerative mucositis that had developed after seven days of acetic acid application.

**Results:**

Chrysin gel after 7 days reduced buccal ulcer and inhibited inflammation and degradation of collagen induced by acetic acid via suppression of MDA, TNF-α, NF-κβ, IL-6, and matrix metalloproteinases (MMP9) as well as stimulation of GSH and vascular endothelial growth factor (VEGF) enhancing healing effect. Histopathological results exhibited that chrysin alleviated the muscle bundle degeneration in the tongue and the acinar lining epithelium degeneration of submandibular salivary glands.

**Conclusion:**

Chrysin gel can be used as an oral gel via its anti-inflammatory activity and induction of VEGF. It can also be tested clinically in oral human ulcers.

## Introduction

The prevalence of oral ulcers (OU), considered one of the most frequent oral mucosal disorders, has steadily risen yearly, impacting up to 30% of the population (1). It causes intense pain that can be exacerbated by physical contact and may even occur spontaneously, significantly reducing the quality of life (2). OUs, which are inflammatory lesions affecting the oral mucosa, can result from various causes, including microbial infections, immune system imbalances, radiation, and hypersensitivity (3). OU presents as a non-specific inflammatory response characterized by cell edema, dissolution, ulceration, and necrosis (4). An easy and economical way to cause oral ulcers is to apply acetic acid to the mandibular alveolar oral mucosa. As a result, we developed a system to evaluate oral mucosal pain in conscious rats (5). Currently, the clinical management of oral ulcers mainly relies on the topical application of antibiotics or corticosteroids to reduce symptoms (6). However, the overuse or misuse of antibiotics can lead to the development of antibiotic-resistant bacteria, posing a significant public health threat (7).

Controlling inflammation and accelerating the healing process are key strategies in treating oral ulcers (8). As a result, most treatments involve anti-inflammatory drugs. Topical application of these drugs is often the preferred delivery method, as it provides targeted relief and minimizes systemic side effects (9). In the wounded area, there is a reduction in vascular endothelial growth factor (VEGF)(10), while during the wound healing process, VEGF is elevated and plays a pivotal role in promoting the activity of tissue factor expression in both endothelial cells and monocytes. VEGF is also crucial for inducing angiogenesis and enhancing microvascular permeability, both essential for effective tissue repair and regeneration (11). Within the microenvironment of a wound, matrix metalloproteinases (MMPs) and proteolytic enzymes cleave the extracellular matrix (ECM) and affect growth factors and cytokines, stimulating their cellular movement (12). When MMPs are activated, they are expressed in different cells, including fibroblasts, keratinocytes, and inflammatory cells, in response to cytokine signals and growth factors. MMP-9, in particular, plays a significant role in the angiogenesis process and in promoting keratinocyte migration during wound healing (13).

Natural compounds have garnered significant attention for their ability to cure inflammatory illnesses due to their potent anti-oxidant and anti-inflammatory properties (14). Chrysin (5, 7-dihydroxyflavone) is a beneficial flavonoid in numerous plant gum, propolis, and honey extracts. Chrysin has a variety of positive actions, including anti-oxidant, neuroprotective, cardioprotective, anti-inflammatory, and antidiabetic properties. These effects make chrysin a beneficial substance for treating a variety of health issues like colon cancer (15), brain aging (16), and schizophrenia (17). Its ability to lower inflammation and oxidative stress makes it particularly promising for aiding wound healing through improved tissue repair and regeneration (18). Our study aims to explore the potential role of chrysin in enhancing wound healing by leveraging its potent anti-oxidant and anti-inflammatory properties. The investigation focuses on elucidating the underlying mechanisms of action, particularly the regulation of key molecular pathways such as MMP-9 and VEGF, which are crucial for extracellular matrix remodeling, angiogenesis, and tissue regeneration. 

## Materials and Methods

### Animals

A total of thirty-two Albino male Wistar rats, each weighing between 150 and 170 grams, were sourced from the colony section of the National Research Centre (NRC) in Egypt. The rats were maintained in an environment with regulated temperature and humidity, specifically at 23±2 °C and 45–55% relative humidity. During the study, they were housed individually in sanitized cages and provided unlimited access to tap water and pelleted food. All animals received humane care, and the study protocols were carried out according to the ethical guidelines for the care and use of experimental animals approved by the Medical Research Ethics Committee (MREC) at the NRC (20195).

### Chemicals

Reduced glutathione (GSH) and malondialdehyde (MDA) were purchased from Bio Diagnostic, Egypt.

Nuclear factor kappa B (NF-κB), tumor necrosis factor-alpha (TNF-α), interleukin-6 (IL-6), matrix metalloproteinases (MMP9), and vascular endothelial growth factor (VEGF) enzyme-linked immunosorbent assay (ELISA) kits were purchased from Sunlong Biotech Co., Ltd., China.

### Experimental design

*Induction of oral ulcers*

Ketamine (50 mg/kg) and xylazine (5 mg/kg) were used for rat anesthesia. Subsequently, a circular filter paper measuring 6 mm in diameter was saturated with 15 μl of 50% acetic acid and employed to induce aseptic tissue necrosis. To form round ulcers, the acid-imbued paper was applied to the rat labial gingival tissue for 60 sec (19).

Male Wistar Albino rats (200–250 g) were randomly divided into four groups, with eight rats in each group. The first group, Normal control rats, received blank gel. The second group (positive control group), oral ulcer rats, received acetic acid. The third and fourth groups: Rats received chrysin gel (1% and 2%) (20) for 7 days, 24 hr after acetic acid application (21).

### Biochemical indices

Buccal tissue samples were collected from different groups of rats seven days post-ulcer. A homogenizer (Medical Instruments, MPW-120, Poland) was utilized to create 10% (w/v) homogenates in phosphate buffer at pH 7.4. The homogenates were then centrifuged for 10 min at 1000 rpm and 4 °C in a cooling centrifuge (2 k15, Sigma, Germany) to remove cell debris (22). Supernatants were collected to evaluate the buccal content of TNF-α, NF-κβ, IL-6, MMP9, and VEGF (23).

### Histopathological examination of buccal samples

Buccal samples were collected from each rat across various groups and preserved in 10% formal saline for 24 hr. Following this, tap water was used to rinse the samples. Furthermore, a series of alcohol dilutions were employed for dehydration. After being cleaned with xylene, the samples were embedded in paraffin and heated to 56 degrees Celsius for 24 hr in a hot air oven. Paraffin wax tissue blocks were subsequently prepared for sectioning at a thickness of 4 microns using a rotary LEITZ microtome. The resulting tissue sections were placed on glass slides, deparaffinized, and stained with hematoxylin and eosin for examination under a light electric microscope (24).

### Statistical analysis

For all quantitative comparisons in our study, we employed one-way analysis of variance (ANOVA), supplemented by Tukey’s multiple comparisons test (GraphPad Prism 8.0, USA). The findings are presented as the mean ± SEM based on six rats, with a significance threshold set at *P*-value≤0.05.

## Results

### Effect of chrysin on oral ulcer morphology

**Figure 1:**
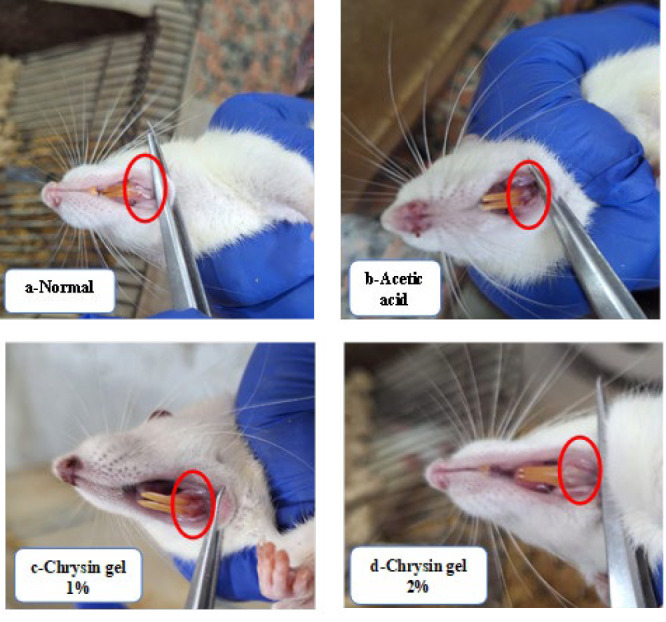
Macroscopic morphology of the oral healing progress of chrysin gel in Albino Wistar rats

The untreated group showed a non-healed oral ulcer after 7 days, whereas the topical application of chrysin gel (1% & 2%) for 7 days enhanced ulcer healing (Figure 1). 

### Effect of chrysin on oxidative stress in oral ulcer model

**Figure 2:**
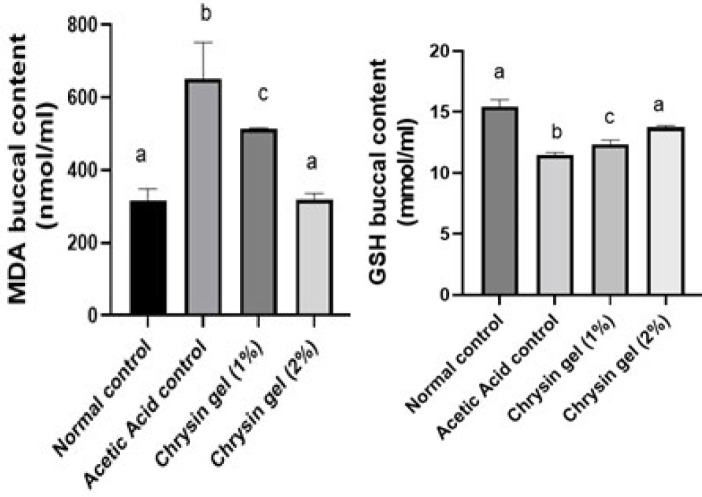
Effect of chrysin gel on MDA and GSH oral ulcer healing in Albino Wistar rats

In an oral ulcer, application of acetic acid elevated MDA and reduced GSH buccal contents by 69% and 26%, respectively, compared with the normal control group. Chrysin oral gel (1% and 2%) application for 7 days decreased buccal contents of MDA by 13% and 48% and increased GSH buccal contents by 8% and 20%, respectively, compared to the ulcer group. In addition, Chrysin oral gel 2% returned MDA and GSH values to normal levels (Figure 2a & b). 

### Effect of chrysin on the expression of TNF- α, NF-κβ, and IL-6 in oral ulcer model

**Figure 3:**
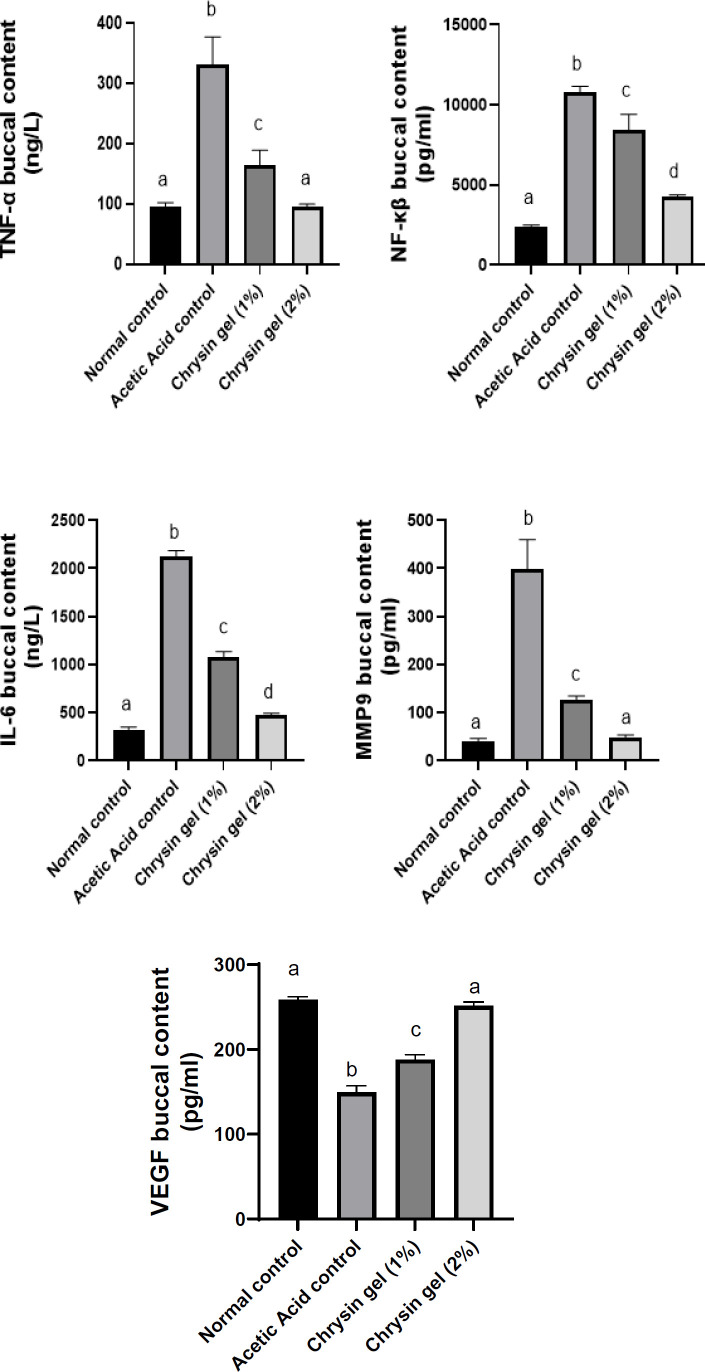
Effect of chrysin gel on the expression of TNF-α, NF-κβ, IL-6, MMP9 and VEGF in oral ulcer healing in Albino Wistar rats

In oral ulcers, the application of acetic acid elevated proinflammatory cytokines such as TNF-α, NF-κB, and IL-6 buccal contents by 246%, 345%, and 558%, respectively, compared with the normal control group. Chrysin oral gel (1% and 2%) application for 7 days decreased buccal contents of TNF-α by 51% and 71%, NF-κβ by 22% and 60%, and IL-6 by 49% and 78%, respectively, as compared to the ulcer group. In addition, Chrysin oral gel 2% returned TNF-α values to normal levels (Figure 3a-c). 

### Effect of chrysin on matrix metalloproteinase

Our results revealed that acetic acid application increased MMP9 buccal contents, inducing oral ulcers by 883% compared with the normal control group. Chrysin oral gel (1% and 2%) application for 7 days reduced MMP9 by 68% and 88%, respectively, compared to the oral ulcer group. In addition, applying chrysin 2 % gel returned MMP9 to its normal value (Figure 3d). 

### Effect of chrysin on cell proliferative marker

The application of acetic acid reduced the buccal contents of VEGF by 42% compared to the normal group; however, treatment with chrysin gel (1% and 2%) increased the contents of VEGF by 26% and 68%, respectively, compared to the ulcer group. In addition, chrysin gel 2% returned VEGF content to its normal level (Figure 3e). 

### Histopathological results of tongue and submandibular salivary glands

**Figure 4:**
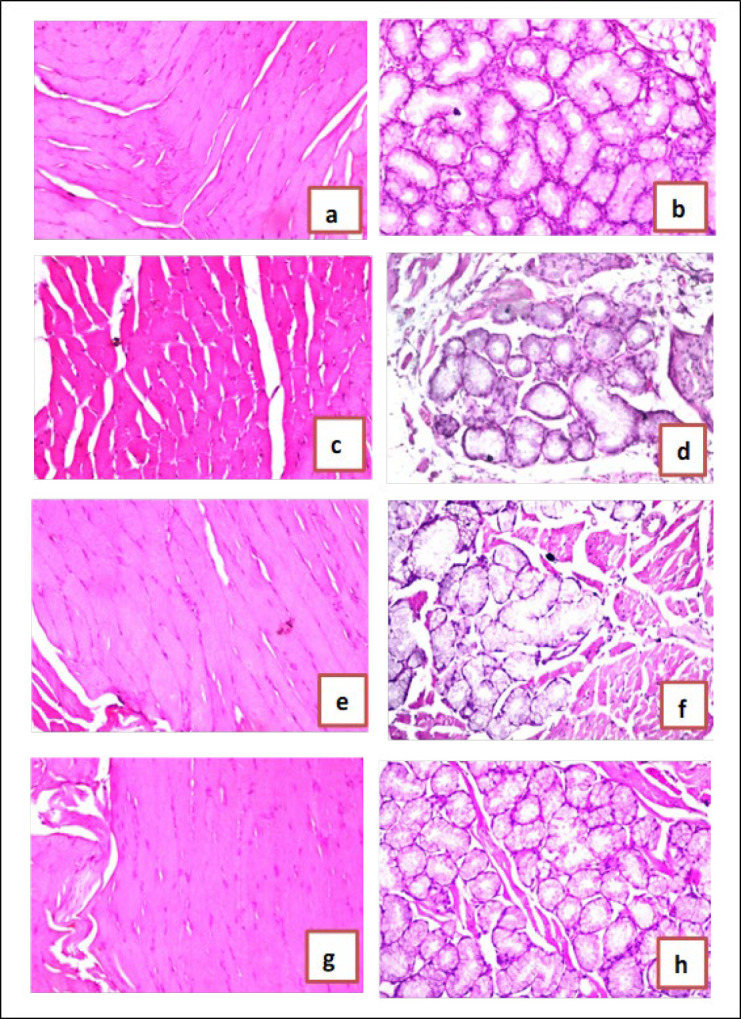
Effect of chrysin gel on buccal tissue in Albino Wistar rats

In the normal control group, there was no histopathological alteration, and the normal histological structure of the muscle bundles was recorded in the tongue (Figure 4a); moreover, there was no histopathological alteration, and the normal histological structure of the acini and surrounding stroma were recorded in submandibular salivary glands (Figure 4b). In the oral ulcer control group, the muscle bundles showed degeneration as recorded in the tongue (Figure 4c). Degenerative change was detected in the acinar lining epithelium of submandibular salivary glands (Figure 4d) In the wounded rats treated with chrysin oral gel (1%) group, there was amelioration in histopathologicalalteration in the muscular layer of the tongue and in the acini of submandibular salivary glands (Figure 4 e & f). In the wounded rats treated with chrysin oral gel (2%) group, there was no histopathological alteration in the tongue’s muscular layer and in the submandibular salivary glands’ acini (Figure 4g & h).

## Discussion

Oral ulcers are a highly prevalent condition affecting the oral mucosa, often becoming chronic or recurrent (25). Managing these ulcers typically requires long-term use of medications, including local corticosteroids, antiseptics, anti-inflammatory agents, and local anesthetics (26). However, these treatments can cause side effects that negatively impact patients’ quality of life (27). The buccal mucosa is the most commonly affected area in the oral cavity, followed by the tongue and the lower lip (28). This study examines the effect of chrysin applied to rats’ inner cheeks and tongues over seven days in conjunction with acetic acid to induce buccal ulceration.

The acetic acid-induced oral ulcer model is frequently utilized as it reliably reproduces the histopathological features associated with oral ulcers (19). Wound healing is a highly intricate biological process the human body performs naturally. It involves tightly regulated stages, including inflammation, cell proliferation, wound contraction, angiogenesis, matrix remodeling, and re-epithelialization (29). When a wound is left untreated, it can result in pain, inflammation, and potential infection (30). During the inflammatory phase, swelling and pain occur as the body arranges the area for healing and immobilizes the wound. This is followed by the fibroblastic phase, where the structural framework is rebuilt, and finally, the remodeling phase restores the tissue to its final form (31). In this study, the topical application of 1% and 2% w/w chrysin demonstrated notable anti-inflammatory effects, evidenced by reduced inflammation levels in the treated group compared to the acetic acid group. Similarly, a prior study on mice with acetic acid-induced gastric ulcers reported that chrysin administration significantly reduced macroscopic lesion areas. This therapeutic effect was linked to a decrease in the expression of inflammatory markers (32). These results demonstrate that chrysin supports ulcer healing by modulating inflammatory responses and promoting tissue repair mechanisms.

In addition to its anti-inflammatory properties, chrysin exhibits potent anti-oxidant activity, which is critical in mitigating oxidative stress associated with ulcer formation. Oxidative stress plays a significant role in the development of ulcers. In contrast, anti-oxidants help mitigate cellular damage caused by the heightened production of reactive oxidative species (33). The present study is the first one that highlights chrysin’s anti-oxidant activity in buccal tissues and is supported by several studies on its role in wound healing (34). Chrysin, a natural flavonoid in various plants, exhibits significant anti-oxidant properties. Its anti-oxidant activity is attributed to its chemical structure, which enables it to scavenge free radicals and reduce oxidative stress (35). Anti-oxidants are believed to significantly contribute to the acceleration of wound healing by alleviating inflammation in the oral mucosa, which in turn lowers the likelihood of developing precancerous lesions (36). This offers additional understanding of the healing benefits of chrysin. It also reinforces the noted anti-inflammatory effects, given the relationship between oxidative stress and inflammation. In line with our findings, chrysin has been shown to enhance anti-oxidant defenses by increasing GSH levels and reducing MDA concentrations. For instance, a previous study demonstrated that in lipopolysaccharide-induced sepsis in rats, chrysin treatment increased the activities of anti-oxidant enzymes, such as catalase (CAT), glutathione peroxidase (GSH-Px), and superoxide dismutase (SOD), while decreasing MDA levels, further supporting its anti-oxidative efficacy (37). These findings highlight the importance of anti-oxidants in accelerating the healing of oral ulcers and improving patient outcomes.

The produced ROS trigger the restoration of transcription factors that promote the expression of proinflammatory mediators, including TNF-α and IL-6 (38). Previous studies have indicated that elevated levels of proinflammatory cytokines, including TNF-α and IL-6, lead to a reduced healing rate. This reduction is attributed to the enhancement of apoptosis and a decline in fibroblast mobility (39). NF-κB activation plays an integral role in the inflammatory response by inducing the release of a wide range of proinflammatory cytokines, including TNF-α and IL-6. As a transcription factor, NF-κB is an important regulator of the immune response and is elaborated in the promotion of oxidative stress, further exacerbating inflammation and impeding the wound-healing process (40). The topical application of chrysin significantly reduced the levels of IL-6 and TNF-α in acetic acid-induced oral ulcers. Chrysin’s anti-inflammatory effects could be attributed, in part, to its ability to suppress NF-κB activation. By inhibiting NF-κB, chrysin likely prevents the excessive production of inflammatory cytokines and oxidative stress, thus enhancing the healing process. This mechanism of action is consistent with findings from other studies where chrysin has been shown to modulate inflammatory pathways and promote tissue repair by reducing NF-κB-driven inflammation. These findings align with existing research suggesting that chrysin exhibits strong anti-inflammatory properties on rats’ amiodarone extravasation-induced skin injury model by decreasing TNF-α and IL-6 levels (41). Similarly, chrysin has demonstrated anti-inflammatory effects in models of spinal cord injury by suppressing NF-κB activation, thereby reducing the expression of proinflammatory cytokines and promoting tissue repair (42). Chrysin also modulates the TLR4/NF-κβ pathway, ameliorating diabetes in rats (43). Therefore, chrysin’s ability to reduce NF-κB activation and subsequently lower cytokine levels, such as IL-6 and TNF-α, is a crucial aspect of its therapeutic potential in managing oral ulcers and other inflammatory conditions.

Further to the findings of this study, TNF-α plays a critical role in wound healing by triggering the release of matrix metalloproteinases (MMPs), which leads to the degradation of matrix proteins and growth factors, thus delaying the healing process (44). MMP-9, in particular, is elaborated in the degradation of ECM components and contributes to the migration of keratinocytes, which is essential for wound healing (45). Furthermore, TNF-α enhances the synthesis of proangiogenic factors, including the vascular endothelial growth factor (VEGF), which promotes angiogenesis and aids in wound healing (46). However, an overactive inflammatory response, characterized by excessive MMP-9 and TNF-α, can hinder proper healing by degrading the ECM and promoting unnecessary inflammation.

In the present study, chrysin administration effectively decreased the elevated levels of MMP-9 and significantly increased VEGF levels, suggesting its role in promoting tissue repair. This aligns with a previous study where chrysin has been shown to mitigate diabetic foot ulcers by enhancing VEGF expression, thus supporting angiogenesis and improving the wound healing process (47). In another study, chrysin enhances the release of nerve growth factor (NGF), treating brain aging (48). So, chrysin can regulate MMP-9 levels and boost VEGF, highlighting its potential to promote a balanced inflammatory response and facilitate tissue regeneration, which is crucial for effective wound healing. These findings further underscore the therapeutic potential of chrysin in managing chronic wounds and enhancing tissue repair processes.

Data are presented as the mean ± SD (n=8) for each group. Statistical analysis was conducted by ANOVA followed by Tukey’s multiple comparisons test. Same letter means non-significant difference, while different letter means significant difference at *P*<0.05. MDA: Malondialdehyde; GSH: Glutathione

Data are presented as the mean ± SD (n=8) for each group. Statistical analysis was conducted by ANOVA followed by Tukey’s multiple comparisons test. Same letter means non-significant difference, while different letter means significant difference at *P*<0.05.

Histological picture of the group of rats kept as control (a) there was no histopathological alteration and the normal histological structure of the muscle bundles were recorded in. Group of control group rats (b) there was no histopathological alteration and the normal histological structure of the acini and surrounding stroma were recorded in. Group of wounded rats treated by acetic acid (c) the muscle bundles showed degeneration as recorded in. Group of wounded rats treated by acetic acid (d) degenerative change was detected in the acinar lining epithelium. Group of wounded rats and chrysin oral gel (1%) (e,f) there was no histopathological alteration in the muscular layer and in the acini. Group of wounded rats and chrysin oral gel (2%) (g,h) there was no histopathological alteration in the muscular layer and in the acini. H&E pictures from left column is tongue, right column is submandibular salivary glands.

## Conclusion

The current study provides significant data supporting chrysin’s therapeutic potential in treating mouth ulcers and wound healing. Chrysin reduces the levels of proinflammatory cytokines IL-6 and TNF-α, which are known to cause delayed healing. Chrysin inhibits NF-κB activation, promoting tissue healing and lowering oxidative stress. Furthermore, chrysin’s ability to reduce MMP-9 levels and to elevate VEGF levels, suggests that it promotes angiogenesis and facilitates wound healing via better ECM remodeling and keratinocyte migration.

## Data Availability

All relevant data generated or analyzed during this study are included in this article. Further inquiries can be directed to the corresponding author.

## Declaration

We have not used any AI tools or technologies to prepare this manuscript
